# Use of mesenchymal stem cells to enhance or restore fertility potential: a systematic review of available experimental strategies

**DOI:** 10.1093/hropen/hoad040

**Published:** 2023-10-25

**Authors:** L Cacciottola, F Vitale, J Donnez, M M Dolmans

**Affiliations:** Gynecology Research Unit, Institut de Recherche Expérimentale et Clinique, Université Catholique de Louvain, Brussels, Belgium; Gynecology Research Unit, Institut de Recherche Expérimentale et Clinique, Université Catholique de Louvain, Brussels, Belgium; Society for Research into Infertility, Brussels, Belgium; Université Catholique de Louvain, Brussels, Belgium; Gynecology Research Unit, Institut de Recherche Expérimentale et Clinique, Université Catholique de Louvain, Brussels, Belgium; Department of Gynecology, Cliniques Universitaires Saint-Luc, Brussels, Belgium

**Keywords:** stem cell therapy, ovarian reserve, gonadotoxic damage, fertility restoration, ovarian rejuvenation, ovarian tissue transplantation

## Abstract

**STUDY QUESTION:**

To what extent does regenerative medicine with stem cell therapy help to address infertility issues for future clinical application?

**SUMMARY ANSWER:**

Regenerative medicine using different stem cell sources is yielding promising results in terms of protecting the ovarian reserve from damage and senescence, and improving fertility potential in various preclinical settings.

**WHAT IS KNOWN ALREADY:**

Regenerative medicine using stem cell therapy is emerging as a potential strategy to address a number of issues in the field of human reproduction. Indeed, different types of adult and fetal mesenchymal stem cells (MSCs) have been tested with promising results, owing to their ability to differentiate into different tissue lineages, move toward specific injured sites (homing), and generate a secretome with wound-healing, proangiogenic, and antioxidant capacities.

**STUDY DESIGN, SIZE, DURATION:**

Guided by the checklist for preferred reporting items for systematic reviews and meta-analyses, we retrieved relevant studies from PubMed, Medline, and Embase databases until June 2023 using the following keywords: ‘mesenchymal stem cells’ AND ‘ovarian follicles’ OR ‘ovarian tissue culture’ OR ‘ovarian follicle culture’ OR ‘cumulus oocyte complex’. Only peer-reviewed published articles written in English were included.

**PARTICIPANTS/MATERIALS, SETTING, METHODS:**

The primary outcome for the experimental strategies was evaluation of the ovarian reserve, with a focus on follicle survival, number, and growth. Secondary outcomes involved analyses of other parameters associated with the follicle pool, such as hormones and growth factors, ovarian tissue viability markers including oxidative stress levels, oocyte growth and maturation rates, and of course pregnancy outcomes.

**MAIN RESULTS AND THE ROLE OF CHANCE:**

Preclinical studies exploring MSCs from different animal origins and tissue sources in specific conditions were selected (n = 112), including: *in vitro* culture of granulosa cells, ovarian tissue and isolated ovarian follicles; ovarian tissue transplantation; and systemic or intraovarian injection after gonadotoxic or age-related follicle pool decline. Protecting the ovarian reserve from aging and gonadotoxic damage has been widely tested *in vitro* and *in vivo* using murine models and is now yielding initial data in the first ever case series of patients with premature ovarian insufficiency. Use of MSCs as feeder cells in ovarian tissue culture was found to improve follicle outcomes and oocyte competence, bringing us one step closer to future clinical application. MSCs also have proved effective at boosting revascularization in the transplantation site when grafting ovarian tissue in experimental animal models.

**LIMITATIONS, REASONS FOR CAUTION:**

While preclinical results look promising in terms of protecting the ovarian reserve in different experimental models (especially those *in vitro* using various mammal experimental models and *in vivo* using murine models), there is still a lot of work to do before this approach can be considered safe and successfully implemented in a clinical setting.

**WIDER IMPLICATIONS OF THE FINDINGS:**

All gathered data on the one hand show that regenerative medicine techniques are quickly gaining ground among innovative techniques being developed for future clinical application in the field of reproductive medicine. After proving MSC effectiveness in preclinical settings, there is still a lot of work to do before MSCs can be safely and effectively used in different clinical applications.

**STUDY FUNDING/COMPETING INTEREST(S):**

This study was supported by grants from the Fonds National de la Recherche Scientifique de Belgique (FNRS-PDR T.0077.14, FNRS-CDR J.0063.20, and grant 5/4/150/5 awarded to Marie-Madeleine Dolmans), Fonds Spéciaux de Recherche, and the Fondation St Luc. None of the authors have any competing interest to disclose.

**REGISTRATION NUMBER:**

N/A.

WHAT DOES THIS MEAN FOR PATIENTS? ‘Regenerative medicine’ describes a potential clinical approach for managing various pathological conditions (i.e. diseases or injury) that have no current treatment options, including many in the field of human reproduction. The techniques involved are based on the use of mesenchymal stem cells (MSCs), which are non-specialized cells that can give rise to infinitely more cells of the same type, as well as other cell types. Stem cells have the potential to regenerate and produce signals that promote wound healing (i.e. tissue regeneration) in different organs. For this reason, various experimental strategies are under development to exploit the ability of stem cells to protect or restore fertility. More specifically, stem cells may help protect the ovary (and the follicles/eggs it contains) against different types of injury, caused either by the aging process or use of chemotherapy after a cancer diagnosis. Both conditions significantly decrease a woman’s fertility and chance of pregnancy. Would MSC infusion or localized therapy help repair damaged ovaries? We undertook a careful review of all studies that investigated any strategy using MSCs in either animal models or human studies, to provide evidence that MSCs could improve fertility outcomes. Studies that evaluated ‘ovarian reserve’—that is the reproductive potential left within a woman’s two ovaries based on number and quality of eggs—were our primary interest. The results showed that different types of MSCs have been tested in attempts to enhance fertility in various contexts. Among these, they improve follicle survival and growth, and are also able to reverse chemotherapy-induced ovarian damage and improve follicle pool survival by boosting ovarian re-growth of blood vessels. Based on recently gathered data, studies in regenerative medicine are yielding encouraging results in terms of restoring fertility. However, work is still needed to optimize techniques and test their safety before they can become available to patients.

## Introduction

Regenerative medicine is emerging as a potential tool to manage various pathological conditions that have no current treatment. There is particular interest in reproductive medicine, since the number of ovarian follicles, which are the main functional units of the ovary and responsible for female fertility, is finite at birth and continues to fall during the reproductive lifespan, with no ability to regenerate (Dolmans *et al.*, 2021a). Moreover, follicles, and especially the oocytes contained within, are characterized by specific damage repair mechanisms, as their key role is to convey undamaged genetic information to future offspring (Maidarti *et al.*, 2020). This makes follicles particularly vulnerable to potentially damaging effects of various stimuli, resulting in reduced fertility potential and premature depletion of the ovarian reserve.

Regenerative medicine techniques are based on use of stem cells, which are defined as cells originating from a multicellular organism that are capable of giving rise to infinitely more cells of the same type (self-renewal), as well as other cell types, by differentiation (potency). They represent populations of non-specialized cells that have the potential to differentiate into specialized cellular subtypes (Weissman, 2000). Stem cells can be classified according to their origin into embryonic, fetal, adult, and induced pluripotent stem cells (Takahashi and Yamanaka, 2006; Bacakova *et al.*, 2018).

The present review will focus exclusively on fetal and adult stem cells since their use as therapeutic tools is not contentious and is actually considered the most promising for regenerative medicine and tissue engineering purposes. Fetal stem cells can be isolated from various surplus fetal tissues, such as amnion, chorion, amniotic fluid and the umbilical cord, and show greater multilineage differentiation capacity than adult stem cells. Adult, or somatic, stem cells are located in all organs and tissues to varying degrees, with the function of maintaining and repairing them (Ding *et al.*, 2011). Most of them are multipotent, with cell lineage-specific restrictions, or oligo/unipotent, also known as progenitor cells (Melchiorri *et al.*, 2016). Some, like MSCs, are even able to express multipotency toward other cell lineages in specific conditions (Bacakova *et al.*, 2018).

**Figure 1.**
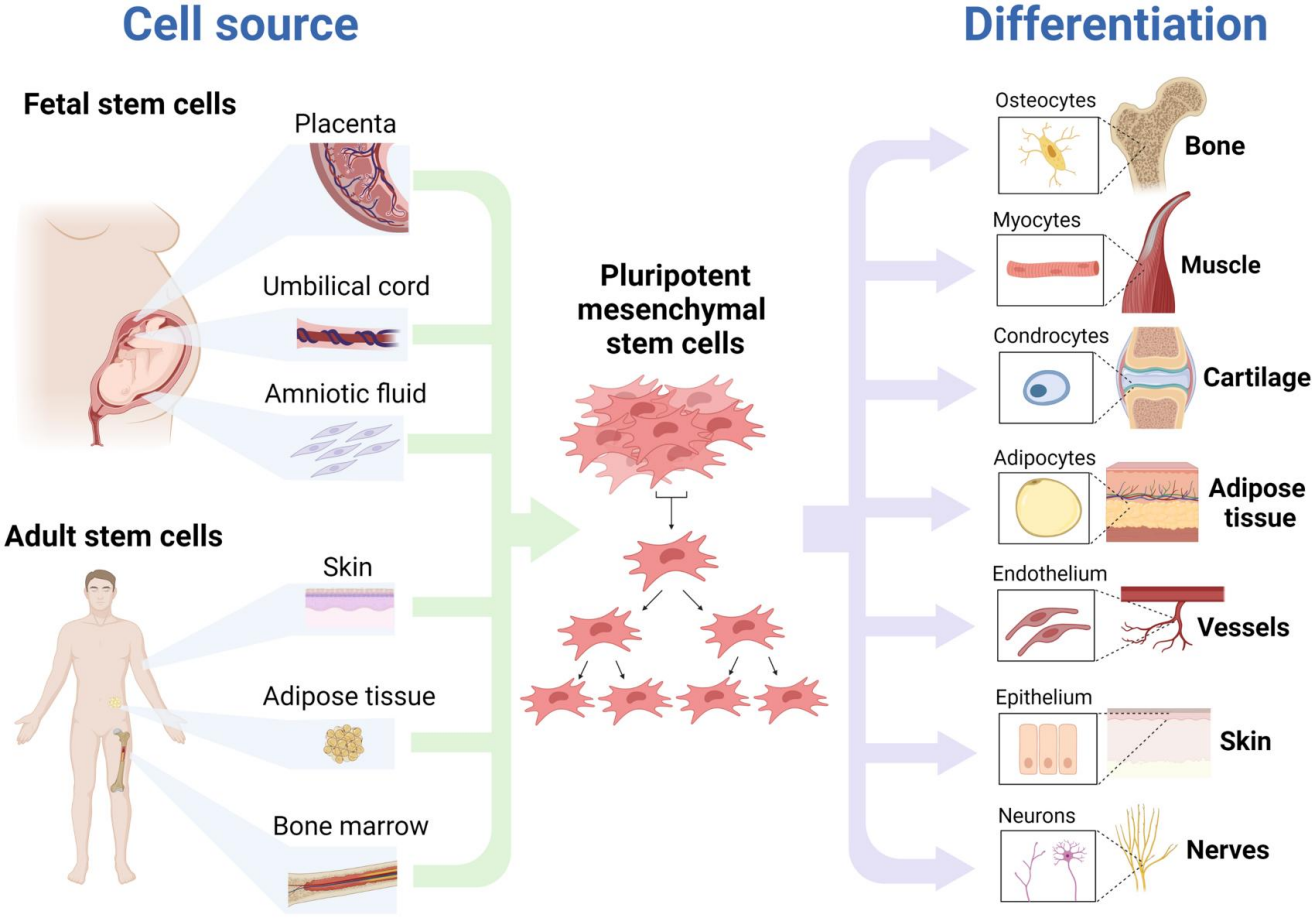
**Sources of mesenchymal stem cells and their capacity for differentiation.** Fetal (placenta, umbilical cord, amniotic fluid) and adult (skin, adipose tissue, bone marrow) sources of pluripotent mesenchymal stem cells are able to grow *in vitro* and differentiate into different tissues, including bone, muscle, cartilage, adipose tissue, vessels, skin, and nerves. Unique populations of MSCs by their multilineage differentiation capacity, facility to grow as adherent cells in standard culture conditions, and ability to express specific marker profiles.

MSCs are a heterogeneous population of cells with multilineage differentiation capacity (Bacakova *et al.*, 2018). They grow *in vitro* as plastic-adherent cells with a fibroblast-like shape, and organize themselves into colonies (Dominici *et al.*, 2006). The International Society for Cellular Therapy established certain criteria to identify unique populations of MSCs by their multilineage differentiation capacity, facility to grow as adherent cells in standard culture conditions, and ability to express specific marker profiles, namely CD90, CD73, CD105, and MHCI, but not CD14, CD34, CD45, CD31, or MHCII (Dominici *et al.*, 2006) (Fig. 1). They can be easily isolated from a number of fetal and adult tissues, the former including amniotic fluid-derived MSCs (AF-MSCs), umbilical cord-derived MSCs (UC-MSCs), and placenta-derived MSCs (PD-MSCs), and the latter including bone marrow-derived MSCs (BM-MSCs), adipose tissue-derived MSCs (AT-MSCs), skin-derived MSCs (S-MSCs), and even menstrual blood-derived MSCs (Men-MSCs) (Kern *et al.*, 2006; Polonio *et al.*, 2020).

The impact of MSCs appears to depend on their capacity to secrete a diversity of cytokines, chemokines, and growth factors. Some of these secreted factors play a crucial role in controlling cell proliferation and apoptosis rates, thereby promoting regeneration of injured tissues (Wang *et al.*, 2011). MSCs also exert a modulatory effect on the immune system (Wei *et al.*, 2013), suppressing excessive responses by macrophages, dendritic cells, and natural killer cells through cell-to-cell contact and release of soluble immunosuppressive factors (Uccelli *et al.*, 2008). They also possess homing properties, namely the capacity to directionally migrate to distant damaged organs/tissues in response to signaling molecules (Moser and Loetscher, 2001). These abilities have fostered growing interest in the field of regenerative medicine based on the idea that MSC infusions or localized therapy may well aid organ and tissue repair.

Increasing evidence of the potential of MSCs to treat different diseases is currently being gathered to facilitate their transition from bench to bedside. Different disease models are being tested and numerous clinical trials are ongoing (Rodríguez-Fuentes *et al.*, 2021). While significant progress has been made, stem cell therapy is still several steps away from use in clinical practice. One of the main issues is standardizing the methodology to isolate, characterize, and expand MSCs before their clinical application. This is not always easy, as it may involve different MSC subpopulations that could later show heterogeneous behavio*r in vitro* (Baer and Geiger, 2012). Such heterogeneity is contingent on multiple factors, such as donor characteristics (age, gender, BMI, ethnicity, pre-existing conditions, and pathologies) (Baer and Geiger, 2012), isolation protocols (different storage temperatures and isolation times), and the flow cytometry protocol applied for cell sorting (Griesche *et al.*, 2010). Another challenge is MSC safety in each particular experimental model. Indeed, risks may be related to the microbiological safety and genetic stability of MSCs after isolation and expansion. There is also potential for adverse events after *in vivo* use, including concerns about oncogenic safety and control of the host’s immune response to MSCs.

Different types of MSCs have been tested in attempts to enhance fertility in various contexts. Protecting the ovarian reserve from aging and gonadotoxic damage and restoring fertility with strategies like *in vitro* culture and ovarian tissue transplantation remain paramount.

## Materials and methods

The aim of this review was to provide evidence of and information on use of MSCs to improve fertility outcomes. We explored MSCs from different animal origins and tissue sources in specific conditions, including: *in vitro* culture of granulosa cells (GCs), ovarian tissue, and isolated ovarian follicles; ovarian tissue transplantation; and systemic or intraovarian injection after gonadotoxic or age-related follicle pool decline. To this end, we took a systematic approach, reviewing all papers that investigated any of these strategies in either animal models or human studies. The primary outcome was evaluation of the ovarian reserve, with a focus on follicle survival, number, and growth. Secondary outcomes involved analyses of other parameters associated with the follicle pool, such as follicle-related markers like hormones and growth factors, ovarian tissue viability-linked markers like oxidative stress levels, oocyte growth and maturation rates and, of course, pregnancy outcomes.

**Figure 2.**
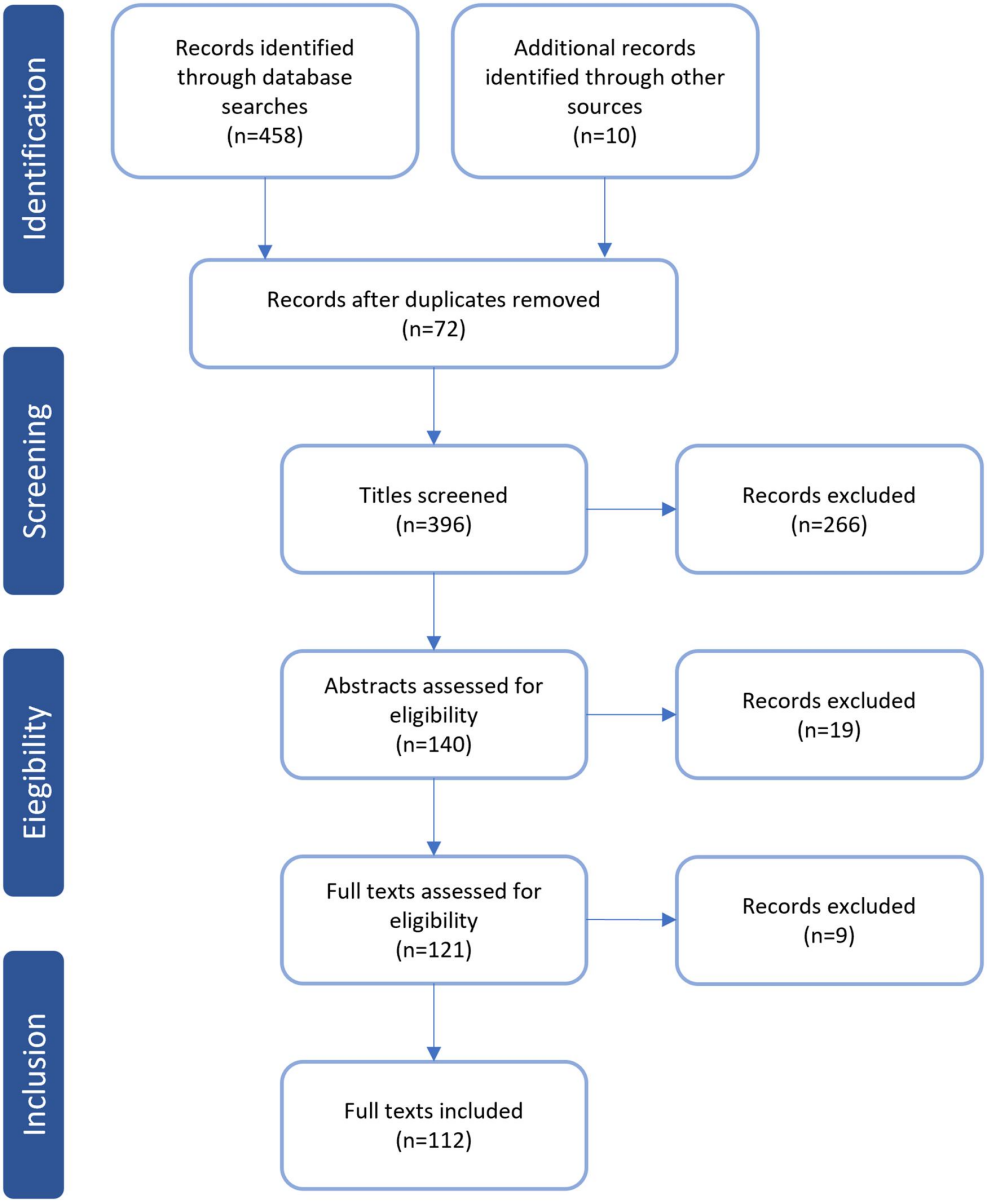
**PRISMA flow diagram of literature search to June 2023.** Literature search methodology for publications investigating use of mesenchymal stem cells in reproductive medicine to enhance follicle outcomes. PRISMA, preferred reporting items for systematic reviews and meta-analyses.

In line with preferred reporting items for systematic reviews and meta-analyses (PRISMA) guidelines (Moher *et al.*, 2009), we conducted a PubMed search up to June 2023 using the following keywords for our research: ‘mesenchymal stem cells’ AND ‘ovarian follicles’ OR ‘ovarian tissue culture’ OR ‘ovarian follicle culture’ OR ‘cumulus oocyte complex’ (458 records). Only peer-reviewed published articles written in English were taken into account. First, all selected studies were imported using Zotero software and duplicates were erased (396 records). Articles were then screened based on their titles (140) and abstracts (121) according to the relevant criteria. Ten more papers were chosen from the references, since they met the same benchmark. After reading the full texts of acquired articles, those fulfilling the required criteria were included (112) (Fig. 2). Ethics approval was not needed because this study did not involve any experimental research. All research data were obtained from published papers.

## Results

### Use of MSCs in different experimental models

#### MSC sources, route of administration and cell labeling

The two most commonly used sources of MSCs in the field of reproductive medicine are UC-MSCs (44 studies) and BM-MSCs (38 studies). Other sources include AT-MSCs (22 studies), Men-MSCs (8 studies), AF-MSCs (7 studies), P-MSCs (7 studies), and S-MSCs (2 studies). Various animal models, including murine, ovine, equine, and human, have been applied to isolate and expand MSCs following different experimental designs. Since *in vivo* administration of MSCs has been performed both locally (by intraovarian injections) and systemically (by i.v. or i.p. injection), a number of authors set out to investigate MSC homing capacities to different organs. To track injected MSCs, they implemented various approaches, including: viral transfection with green fluorescent protein in six studies (Fu *et al.*, 2008; Sun *et al.*, 2013; Deng *et al.*, 2021; Pan *et al.*, 2021; Zhang *et al.*, 2021a; Luo *et al.*, 2022); fluorescent staining of cell membranes, with either PKH26 dye in nine studies (Zhu *et al.*, 2015; Ling *et al.*, 2017, 2019, 2022a,b; Liu *et al.*, 2019; Yang *et al.*, 2019a; Feng *et al.*, 2020; El-Derany *et al.*, 2021) or 1,1′-dioctadecyl-3,3,3′,3′-tetramethyl indocarbocyanine perchlorate (Dil) in two studies (Zarbakhsh *et al.*, 2019; Zhang *et al.*, 2022a); immunohistochemical detection of 5-bromo-2'-deoxyuridine (BrdU)-labeled cells in one study (Besikcioglu *et al.*, 2019); and biocompatible organic fluorescent nanoparticles (LuminiCell Tracker™ 540) also in one study (Salvatore *et al.*, 2021). These labeling methods do not interfere with MSC behavior *in vitro*, as they do not induce cell death nor increased proliferation.

In all cases, MSCs were found in ovarian stroma surrounding the follicles and never inside follicles, indicating that they are unable to differentiate into GCs or oocytes. All staining methods proved effective and labeled MSCs remained in the ovaries for up to 4 weeks (Fu *et al.*, 2008; Sun *et al.*, 2013; Zarbakhsh *et al.*, 2019; Feng *et al.*, 2020; El-Derany *et al.*, 2021; Pan *et al.*, 2021; Salvatore *et al.*, 2021), 6 weeks (Zhu *et al.*, 2015), and 8 weeks (Ling *et al.*, 2017, 2019), irrespective of administration mode. Cell tracking inside the ovaries after systemic administration demonstrated MSC homing capacities toward distant damaged sites (Ling *et al.*, 2017; Besikcioglu *et al.*, 2019; Yang *et al.*, 2019a; El-Derany *et al.*, 2021; Pan *et al.*, 2021; Salvatore *et al.*, 2021). It also showed persistence of MSCs over time, after local ovarian administration proved that they can be retained inside tissue without being cleared by the immune system (Ling *et al.*, 2019; Zarbakhsh *et al.*, 2019). Some authors also investigated their homing capacity toward other distant sites, beyond the ovaries, 24 h after injection, identifying some MSCs in distant organs such as the uterus, spleen, brain, lung, liver, and kidney after both intraovarian (Zhu *et al.*, 2015; Ling *et al.*, 2019; Zhang *et al.*, 2021a) and i.v. injection (Zhu *et al.*, 2015; El-Derany *et al.*, 2021; Salvatore *et al.*, 2021). One research group explored the possibility of increasing homing capacities toward damaged sites using MSCs pretreated by low-intensity pulsed ultrasound (LIPUS) (Ling *et al.*, 2017, 2022a). MSC migration was found to increase both *in vitro* and *in vivo* in the presence of specific molecular signals, such as stromal cell-derived factor 1 (SDF-1), which is enhanced in damaged organs as well as by LIPUS. SDF-1 is a member of the chemokine family, able to drive cell homing through its link to the CXC4 receptor and activation of signals such as phosphatidylinositol 3-kinase/protein kinase B (PI3K/Akt) (Ling *et al.*, 2022a,b).

#### Different indications of MSC derivatives: exosomes and the secretome

Twenty-six experimental designs included use of MSC derivatives, 17 of which involved MSC-derived exosomes (Sun *et al.*, 2017, 2019; Huang *et al.*, 2018; Yang *et al.*, 2019a, 2020a,b; Ding *et al.*, 2020; Zhang *et al.*, 2020, 2021b, 2023; Deng *et al.*, 2021; Li *et al.*, 2021; Geng *et al.*, 2022; Qu *et al.*, 2022; Park *et al.*, 2023) or extracellular vesicles sorted according to different average sizes (Cao *et al.*, 2023; Eslami *et al.*, 2023) and nine conditioned medium containing the MSC secretome (Jia *et al.*, 2017; Damous *et al.*, 2018; Maldonado *et al.*, 2018; Bezerra *et al.*, 2019; Hong *et al.*, 2020; Lee, 2021; Park *et al.*, 2021; Zhang *et al.*, 2021a; Mi *et al.*, 2022). Such approaches are of particular interest as they circumvent some of the issues related to stem cell therapy, including safety and reproducibility of cell line behavior *in vivo*.

Extracellular vesicles include exosomes, macrovesicles and apoptotic bodies, according to their different origin and size. Exosomes are small (40–100 nm diameter) membrane-bound vesicles secreted by cells after invagination of the plasma membrane, before being released into the extracellular space. They can contain proteins, such as cytokines and growth factors, as well as microRNAs (miRNAs) produced by stem cells for paracrine communication purposes, executing comparable functions to their cells of origin in various *in vitro* and *in vivo* experimental models. Use of exosomes derived from MSCs has yielded useful information on follicle behavior. Indeed, GCs were able to take in MSC-derived exosomes (Huang *et al.*, 2018), and this ability was apparently maintained *in vitro* after exposure to chemotherapy (CHT) (Sun *et al.*, 2017; Zhang *et al.*, 2020) and *in vivo* after systemic administration, followed by homing of extracellular vesicles toward damaged ovaries (Eslami *et al.*, 2023). In studies comparing the impact of exosome versus MSC administration, no difference was encountered in follicle outcomes or restoration of hormone levels (Yang *et al.*, 2020a; Zhang *et al.*, 2020; Eslami *et al.*, 2023). One study did, however, detect slightly higher and longer-lasting beneficial effects on the ovarian follicle pool after stem cell injection than exosome administration (Park *et al.*, 2023).

Conditioned medium was mainly used in models of ovarian tissue culture, probably to overcome the difficulties related to different growth rates and metabolic needs of MSCs and ovarian tissue *in vitro*. Discarded medium is richer than isolated exosomes, as it also contains the entire free protein component constituting the secretome. It may, however, also contain discarded solutes from MSC metabolism, which could hamper the overall effect on follicle survival and growth. The only study directly comparing the impact of MSC-derived medium and MSCs themselves was performed on porcine cumulus–oocyte complexes (COCs) and no significant difference was observed in terms of oocyte maturation or embryo development (Lee, 2021). This demonstrated that, at least in this particular model of *in vitro* culture, the two methods are equally effective.

#### Role of miRNAs in ovarian function restoration

Eight papers investigated the role of specific miRNAs as effectors of MSC signaling to GCs (Fu *et al.*, 2017; Sun *et al.*, 2017, 2019; Ding *et al.*, 2020; Yang *et al.*, 2020a,b; Geng *et al.*, 2022; Qu *et al.*, 2022). miRNAs are small non-coding RNAs, displaying regulatory functions to control fundamental effector proteins in cellular function (Memczak *et al.*, 2013). They are increasingly emerging as key players in a number of pathological conditions, including inflammation and cancer (Aljubran and Nothnick, 2021). Their regulatory impact on specific targets makes them attractive as potential therapeutic tools (Rupaimoole and Slack, 2017).

In our selected studies, miRNAs were isolated from different MSC sources, including murine BM (Fu *et al.*, 2017; Sun *et al.*, 2019; Yang *et al.*, 2020a), and human fetal tissues such as UC (Ding *et al.*, 2020; Qu *et al.*, 2022; Sun *et al.*, 2017; Yang *et al.*, 2022b) or AF (Geng *et al.*, 2022).

One study explored the ability of damaged GCs to internalize exosomes and the miRNAs contained within them, and observed an increase in miR-24, miR-106a, miR-19b, and mi-R-25, all related to apoptosis signaling (Sun *et al.*, 2019). A number of studies investigated miRNA content in exosomes derived from human MSCs using large molecular panels and identified miR-17-5p (Ding *et al.*, 2020), miR-664-5p (Sun *et al.*, 2019), miR-369-3p (Geng *et al.*, 2022), and miR-126-3p (Qu *et al.*, 2022) as potential modulators of GC survival and proliferation. MiR-17-5p and miR-126-3p are implicated in regulation of numerous cell activities, including cell cycle progression/arrest (Cloonan *et al.*, 2008; Fang *et al.*, 2015) and PI3K/Akt pathway modulation through interaction with phosphate and tensin homolog (PTEN) (Qu *et al.*, 2022; Geng *et al.*, 2022). Ding *et al.* (2020) proved that this specific miRNA is able to interact with sirtuin gene family, which are key regulators of mitochondrial activity and cell response to oxidative stress. miR-664-5p and miR-369-3p were found to target and downregulate p53, caspase-3 and hypoxia inducible factor 1 α (HIF-1α), potentially having a beneficial effect on ovarian reserve maintenance in the ovary (Sun *et al.*, 2019; Geng *et al.*, 2022). In all studies, exosome administration resulted in an increase in proliferation and a decrease in apoptosis in CHT-damaged human GCs *in vitro*, and enhanced follicle survival in a CHT-damaged murine model *in vivo*.

Expression of specific miRNAs was also modulated *in vitro* by silencing or enhancing in different experimental models, based on literature evidence of their role in ovarian function. Among others, miR-144-5p was investigated, as its expression is associated with an elevated risk of premature ovarian insufficiency (POI) (Kuang *et al.*, 2014). Its silencing *in vitro* was found to revive GCs after CHT-induced damage, through PTEN suppression and dysregulation of the PI3K/Akt pathway, confirming its role as a negative effector of follicle maintenance (Yang *et al.*, 2020a). The same *in vitro* impact was observed by silencing other miRNAs, including miR-146-5p and miR-21-5p (Yang *et al.*, 2020b). As their presence was shown to disrupt follicle growth through PI3K/Akt pathway modulation, their silencing yielded better follicle reserve maintenance (Yang *et al.*, 2020b).

miR-21, on the other hand, was upregulated by lentiviral transfection (Fu *et al.*, 2017). Its overexpression proved effective at counteracting CHT-induced GC apoptosis. Aging mechanisms of action appeared to be involved in modulating the PTEN and PI3K/Akt pathway. This highlights the crucial role that the PI3K/Akt pathway plays in follicle maintenance and growth, and also the challenges of fully understanding how its function is governed.

### Impact of MSCs on ovarian outcomes *in vitro*

MSC secretome properties have also been considered as enhancers of follicle survival and growth in *in vitro* models. Culturing follicles from the primordial stage to fertilizable oocytes is nevertheless a huge challenge (Telfer and Andersen, 2021) and the best approach today involves a multi-step protocol, including: primordial follicle activation and initial growth; follicle development to the antral stage; and oocyte maturation in customized culture conditions to optimize outcomes (McLaughlin *et al.*, 2018). At each step, however, there is significant follicle loss along with follicle growth, and uncertainty about oocyte competence after culture. A number of published studies have investigated the ovarian follicle pool behavior *in vitro* and speculated whether addition of MSCs could enhance follicle outcomes in terms of survival and growth.

#### *In vitro* culture of GCs

In total, 24 studies scrutinized the impact of MSCs on GCs cultured in vitro (Table 1). The most commonly used types (or their exosomes) were murine BM-MSCs (Fu *et al.*, 2008; Guo *et al.*, 2013; Liu *et al.*, 2014a; Chen *et al.*, 2018; Sun *et al.*, 2019; Yang *et al.*, 2020a; Lee, 2021; Eslami *et al.*, 2023; Tian *et al.*, 2023) and human UC-MSCs (Sun *et al.*, 2017; Ding *et al.*, 2020; Hong *et al.*, 2020; Zhang *et al.*, 2020, 2021a; Deng *et al.*, 2021; Li *et al.*, 2021; Qu *et al.*, 2022; Park *et al.*, 2023) in experimental models utilizing murine GCs. Studies using human GCs involved a broader range of MSC sources, including amniotic fluid (Ding *et al.*, 2017; Huang *et al.*, 2020; Geng *et al.*, 2022), adipose tissue (Huang *et al.*, 2018), bone marrow (Park *et al.*, 2021), umbilical cord (Park *et al.*, 2023), and menstrual blood (Yan *et al.*, 2019). In all murine and in two human studies, GCs were exposed to CHT and specifically to alkylating agents, cisplatin or epirubicin, to mimic the gonadotoxic damage *in vitro*. The remaining human studies involved GCs taken from infertile women undergoing oocyte retrieval for IVF, either because of physiological aging (>40 years), a diminished ovarian reserve (DOR or POI), or an aging damage model by exposing them to H_2_O_2_ (Tian *et al.*, 2023).

**Table 1:** Effects of mesenchymal stem cells on granulosa cells *in vitro*.

Author	Mesenchymal stem cells			Granulosa cells		Apoptosis	Proliferation	Other effects
	Animal origin	Tissue source	Preparation used	Animal origin	Chemotherapy			
Chen *et al.* (2018)	Rodent	BM	Heat shock-pretreated cells	Rodent	Phosphoramide mustard	↓		

Deng *et al.* (2021)	Human	UC	Exosomes	Rodent	Cyclophosphamide	↓		↓ Il6 and Il1β

Ding *et al.* (2017)	Human	AF	Cells	Human (DOR and POI)	/	↓	↑	↑ CYP19A1

Ding *et al.* (2020)	Human	UC	Exosomes (10^12^ particles/ml)	Human (POI)	/	↓	↑	↓ SIRT1

Eslami *et al.* (2023)	Human	BM	Cells or extracellular vesicles (20K or 110K)	Rodent	Cyclophosphamide	↓		

Fu *et al.* (2008)	Rodent	BM	Cells	Rodent	Phosphoramide mustard	↓		

Fu *et al.* (2017)	Rodent	BM	Lentivirus-miR-21-transfected cells	Rodent	Phosphoramide mustard	↓		↑ Pi3K/Akt pathway

Geng *et al.* (2022)	Human	AF	Exosomes	Rodent	Cyclophosphamide	↓	↑	↓ YAF2/PDCD5/p53.

Guo *et al.* (2013)	Rodent	BM	Cells	Rodent	Cisplatin	↓		

Hong *et al.* (2020)	Human	UC	Conditioned medium	Rodent	Cisplatin	↓		↑ Pi3K/Akt pathway

Huang *et al.* (2018)	Human	AT	Exosomes	Human (POI and controls)	/	↓	↑	↑ AMH, FSHR, CYP19A1, FOXL2 and SMADs

Huang *et al.* (2020)	Human	AF	Cells	Human (>40 y)	/	↓		↑ AMH, FSHR, CYP19A1 and FOXL2

Li *et al.* (2021)	Human	UC	Exosomes (8–10^10^ particles/ml)	Rodent	Cyclophosphamide	↓	↑	↑ FSHR

Liu *et al.* (2014a)	Rodent	BM	Cells	Rodent	Cisplatin	↓		

Park *et al.* (2021)	Human	BM	Conditioned medium	Human (HGrC1)	Cyclophosphamide	↓	↑	↑ CYP19A1, StAR and FOXL2

Park *et al.* (2023)	Human	UC	1 × 10^7^, 1 × 10^8^, 1 × 10^9^ exosomes	Human (HGrC1)	Cyclophosphamide	↓	↑	↑ CYP19A1, StAR

Qu *et al.* (2022)	Human	UC	Exosomes	Rodent	Cisplatin	↓	↑	↓ PIK3R2

Sun *et al.* (2017)	Human	UC	Exosomes	Rodent	Cisplatin	↓		

Sun *et al.* (2019)	Rodent	BM	Exosomes	Rodent	Cisplatin	↓		

Tian *et al.* (2023)	Human	BM	Cells	Human	H_2_O_2_ (273 mM)		↑	↓ B-Galactosidase, p53 and ROS ↓ N6-methyladenosine

Yan *et al.* (2019)	Human	Men	Cells	Human	Epirubicin	↓	↑	↑ E2, progesterone, AMH, inhibin A and B
Yang *et al.* (2020a)	Rodent	BM	Cells or exosomes	Rodent	Cisplatin	↓		↑ Pi3K/Akt pathway

Zhang *et al.* (2020)	Human	UC	Exosomes	Rodent	Cisplatin	↓		↑ E2 and StAR

Zhang *et al.* (2021a)	Human	UC	Conditioned medium encapsulated in ORP	Rodent	Cisplatin	↓	↑	

AF, amniotic fluid; AMH, anti-Müllerian hormone; AT, adipose tissue; BM, bone marrow; CYP19A1, cytochrome P450 19A1; DOR, diminished ovarian reserve; E2, estradiol; FOXL2, forkhead box L2; FSHR, FSH receptor; HGrC1, human nonluteinized granulosa cell line; Men, menstrual blood; ORP, ovarian reproductive patch; PI3K/Akt, phosphatidylinositol 3-kinase/protein kinase B; p53, protein 53; POI, premature ovarian insufficiency; ROS, reactive oxygen species; StAR, steroidogenic acute regulatory protein; SIRT1, sirtuin 1; UC, umbilical cord.

All studies detected a decrease in GC apoptosis in co-culture with MSCs. Some studies also investigated the impact of co-culture on GC proliferation, either by directly demonstrating increased proliferation rates, or observing activation of signaling pathways, such as PI3K/Akt and Hippo, which are known to be involved in GC survival and proliferation (Fu *et al.*, 2017; Huang *et al.*, 2018, 2020; Hong *et al.*, 2020; Yang *et al.*, 2020a; Li *et al.*, 2021; Park *et al.*, 2021, 2023; Qu *et al.*, 2022). MSC co-culture also appeared to be beneficial for cell hormone function by upregulating markers for steroidogenesis, such as cytochrome P450 19A1 (CYP19A1) and Steroidogenic acute regulatory protein (StAR) (Huang *et al.*, 2018, 2020; Park *et al.*, 2021, 2023; Zhang *et al.*, 2021b), and hormone production, such as estradiol (E2), progesterone, anti-Müllerian hormone (AMH) and inhibin A and B, in the culture medium (Huang *et al.*, 2018, 2020; Yan *et al.*, 2019; Zhang *et al.*, 2021b). MSC co-culture also appeared able to reverse some cellular aging mechanisms, including increased reactive oxygen species (ROS) generation, accumulation of β-galactosidase activity, and elevated methylation of adenosine in mRNA (m6A) in specific genome sites associated with mRNA regulation (Tian *et al.*, 2023).

#### *In vitro* culture of ovarian tissue or isolated follicles

Eighteen studies considered use of different sources of MSCs or their derivatives in ovarian tissue culture (Table 2). Selected animal models were rodents (11 studies), ovine (three studies), and pigs (one study), while four studies used human ovarian tissue. The primary outcome was to determine whether MSCs had a positive impact as ‘feeder cells’ on follicle and/or oocyte culture of: ovine or human ovarian cortical strips (Jia *et al.*, 2017; Hosseini *et al.*, 2019; Arrivabene Neves *et al.*, 2020; Sousa *et al.*, 2021); murine ovaries (Choi *et al.*, 2020; Hong *et al.*, 2020; Buigues *et al.*, 2021a; Cho *et al.*, 2021; Zhang *et al.*, 2021b; Mi *et al.*, 2022; Cao *et al.*, 2023); isolated preantral follicles of murine, ovine, or human origin (Xia *et al.*, 2015; Rajabi *et al.*, 2018; Bezerra *et al.*, 2019; Green *et al.*, 2019; Tomaszewski *et al.*, 2019); and murine or porcine COCs (Maldonado *et al.*, 2018). A positive impact was observed on *in vitro* follicle populations in all studies using rodent ovarian tissue, showing either increased follicle growth or more follicles with a normal morphology. Conflicting conclusions on the role of MSCs in follicle growth were reached for ovine and human ovarian tissue, with some authors detecting a positive effect (Xia *et al.*, 2015; Bezerra *et al.*, 2019; Hosseini *et al.*, 2019; Sousa *et al.*, 2021) and others not (Jia *et al.*, 2017; Arrivabene Neves *et al.*, 2020). Equally controversial was the impact of MSCs on oocyte outcomes after culture. Indeed, two studies found higher meiotic resumption rates in a murine model (Maldonado *et al.*, 2018; Green *et al.*, 2019), while three others, investigating oocyte growth, meiotic resumption, and maturation rates, did not demonstrate any difference compared to *in vitro* culture without MSCs (Rajabi *et al.*, 2018; Bezerra *et al.*, 2019; Arrivabene Neves *et al.*, 2020).

**Table 2:** Impact of mesenchymal stem cells on ovarian tissue *in vitro*.

Author	Mesenchymal stem cells			Ovarian tissue		*In vitro* culture		Follicle and oocyte outcomes	Other biological effects
	Animal origin	Tissue source	Preparation used	Type of culture	Ovarian tissue origin	Culture duration	Cryopreservation		
Arrivabene Neves *et al.* (2020)	Ovine	BM	Cells (1 × 10^3^)	ovarian cortical strips	Ovine	7 days	/	↑ normal follicles =follicle growth =oocyte growth	=GSH activity

Bezerra *et al.* (2019)	Ovine	UC	Conditioned medium	Secondary follicles	Ovine	6 days	/	↑ follicle growth =oocyte meiotic resumption	=GSH activity ↓ ROS ↑ mitochondrial activity

Buigues *et al.* (2021a)	Human	BM and UC	Mobilization from peripheral blood with GCS-F	CHT-treated ovaries (Cy)	Rodent	12and 24 hours	/		↑DNA damage recognition and repair gene expression

Cao *et al.* (2023)	Human	S (iPSCs)	Extracellular vesicles	CHT-treated ovaries (CY)	Rodent	72 hours	/	↑ primordial follicles ↓ follicles apoptosis	↑ PI3K/Akt activation

Cho *et al.* (2021)	Human	P	Cells (1 × 10^4^) +/− VEGF	Ovaries	Rodent	24 and 48 hours	/	↑ follicle growth	↑ VEGF

Choi *et al.* (2020)	Human	P	Cells	Ovaries	Rodent	24, 48 and 72 hours	/		↑ AMH

Green *et al.* (2019)	Rodent	AT	Cells (5 × 10^5^)	Preantral follicles in alginate beads	Rodent	14 days	/	↑ normal follicles ↑ follicle growth ↑ oocyte meiotic resumption	↑ vE2, prog and androstenedione ↑ TZPs between oocytes and GCs

Hong *et al.* (2020)	Human	UC	Conditioned medium	CHT-treated ovaries (cisplatin)	Rodent	6 and 12 hours	/	↓ follicles apoptosis	↑ PI3K/Akt activation

Hosseini et al. (2019)	Human	BM	Cells (3 × 10^5^)	Ovarian cortical strips	Human	8 days	Vitrification	↑ follicle growth ↓ follicle apoptosis	↑ GDF9, FSHR, E2

Jia *et al.* (2017)	Human	UC	Conditioned medium	Ovarian cortical strips	Human	8 days	Vitrification	=primordial follicles ↓ follicle apoptosis	↑microvessel density

Lee (2021)	Human	AT	Cells and conditioned medium	COCs	Porcine	44 hours	/	↑ COC expansion ↓ oocyte apoptosis =fertilization rates	↑ GDF9, BMP15 ↑ VEGF, bFGF, IGF1, IL10 and EGF ↓ ROS

Maldonado *et al.* (2018)	Human	UC	Conditioned medium	COCs	Rodent	24 hours		↑ oocyte maturation rates =fertilization rates	↑ OCT4

Mi *et al.* (2022)	Human	UC	Conditioned medium	Ovaries	Rodent	12 days		↑ follicle activation and growth	↑ FOX03a, DDX4

Rajabi *et al.* (2018)	Human	Men	Cells (5 × 10^3^)	Preantral follicles in alginate beads	Rodent	12 days	+/− vitrification	↑ follicle growth =oocyte maturation rates	↑ GDF9, BMP15, E2 and progesterone
Sousa *et al.* (2021)	Ovine	UC	Cells (1 × 10^4^)	Ovarian cortical strips	Ovine	7 days	/	↑ normal follicles ↑ follicle growth	

Xia *et al.* (2015)	Human	BM	Cells (0.5 × 10^3^ and 0.5 × 10^5^)	Preantral follicles	Human	8 days		↑ follicle growth	↑GDF9, BMP15, E2

Zhang *et al.* (2021b)	Human	Men	Exosomes	Ovaries	Rodent	2, 4 and 6 days	/	↑ follicle growth ↓ follicle apoptosis	↑DAZL, FOXL2

AMH, anti-Müllerian hormone; AT, adipose tissue; bFGF, basic fibroblast growth factor; BM, bone marrow; BMP15, bone morphogenic protein 15; CHT, chemotherapy; COCs, cumulus–oocyte complexes; DAZL, deleted in azoospermia like; E2, estradiol; EGF, epithelial growth factor; FOXL2, forkhead box L2; FOXO3a, forkhead box 03a; FSHR, FSH receptor; GDF9, growth differentiation factor 9; GSH, glutathione; HGF, hepatocyte growth factor; IGF1, insulin growth factor 1; Men, menstrual blood; OCT4, octamer-binding transcription factor 4; P, placenta; PEG, polyethylene-glycol; PI3K/Akt, phosphatidylinositol 3-kinase/protein kinase B; ROS, reactive oxygen species; TFGβ, transforming growth factor β; TZPs, transzonal projections; UC, umbilical cord; VEGF, vascular endothelial growth factor.

These discrepant results may be explained by several factors related to the high variability of the experimental design, not only in the choice of MSC source and ovarian tissue model, but also in the number of cells used for each experiment. Indeed, it is important to note that studies demonstrating a less significant impact of MSCs on ovarian tissue culture are also those using the smallest number of MSCs, for example, 1 × 10^3^ cells (Rajabi *et al.*, 2018; Arrivabene Neves *et al.*, 2020), or MSC-conditioned medium (Jia *et al.*, 2017; Bezerra *et al.*, 2019), which may be insufficient to significantly affect oocyte growth and maturation *in vitro*. Various other markers of ovarian tissue viability have also been assessed, suggesting a positive effect when the MSC secretome is added to *in vitro* culture. These include: an increase in oocyte-related growth factors like growth differentiation factor 9 (GDF) and bone morphogenic protein 15 (BMP15) (Xia *et al.*, 2015; Rajabi *et al.*, 2018; Hosseini *et al.*, 2019; Lee, 2021); enhanced steroidogenesis (Xia *et al.*, 2015; Rajabi *et al.*, 2018; Green *et al.*, 2019; Hosseini *et al.*, 2019); greater production of growth factors, such as basic fibroblast growth factor (bFGF), hepatocyte growth factor (HGF), transforming growth factor β (TFGβ), insulin growth factor 1 (IGF1), vascular endothelial growth factor (VEGF), and epithelial growth factor (EGF) (Tomaszewski *et al.*, 2019; Cho *et al.*, 2021; Lee, 2021); and finally decreased ROS generation *in vitro* (Bezerra *et al.*, 2019; Lee, 2021).

Ovaries were pretreated with chemotherapeutic drugs (cyclophosphamide and cisplatin) in three studies (Hong *et al.*, 2020; Buigues *et al.*, 2021a; Cao *et al.*, 2023). As in previous reports on CHT-treated GCs, the main outcome was to assess whether MSCs protect against gonadotoxic damage. Buigues *et al.* (2021a) used human blood containing either BM-MSCs from both patients with POI after granulocyte colony-stimulating factor (GCS-F) treatment or UC-MSCs from newborn girls. Both MSC sources showed higher levels of expression of genes involved in DNA damage recognition and repair after CHT-induced injury. Hong *et al.* (2020) evidenced that use of human UC-MSCs in CHT-injured rodent ovaries exerted a positive effect by reducing follicle apoptosis and boosting the PI3K/Akt pathway for greater activation and survival.

### Repairing ovarian damage with MSCs

Among the numerous strategies under development to identify an effective treatment for women affected by POI, use of MSCs has been gaining ground over recent years. The goal of stem cell therapy for ovarian rejuvenation is to protect the pool of remaining quiescent follicles still residing in some patients in order to improve their reproductive chances (Polonio *et al.*, 2020). This potential treatment is the result of a number of studies in mice whose ovarian function was damaged by various chemotherapeutic treatments or other toxins, such as ozone. Ovarian injury models have been indeed widely used in research to mimic both infertility and irreversible ovarian failure caused by gonadotoxic drugs depending on dose and mode of administration (Generoso *et al.*, 1971). The impact of MSCs on the remaining ovarian reserve has also been investigated using animal models of natural aging (Polonio *et al.*, 2020).

#### Natural aging and chemotherapy-induced damage: comparing experimental models

Thirteen studies used natural aging animal models for comparison with young and fertile subjects (Table 3). The majority involved use of rodents (Guo *et al.*, 2013; Li *et al.*, 2017; Ding *et al.*, 2018; Huang *et al.*, 2020; Kim *et al.*, 2020; Yang *et al.*, 2020b; Liu *et al.*, 2021; Wang *et al.*, 2022; Zhang *et al.*, 2022a), while three utilized bovines (Malard *et al.*, 2020), mares (Grady *et al.*, 2019), and macaques (Tian *et al.*, 2021). Various sources of MSCs were used at different concentrations. Six of the studies employed a single MSC injection, while the rest opted for repeated administration.

**Table 3:** Studies using mesenchymal stem cells in models of ovarian aging.

Author	Mesenchymal stem cells		Experimental model			Follicle and oocyte outcomes	Hormone activity	Other biological effects
	Stem cell origin	Stem cell source	Animal model	Stem cell use	Time-points			
Ding *et al.* (2018)	Human	AT	Mice (12–14 months)	Repeated injections (1 × 10^7^ cells) for 4 weeks	4 weeks	↑ normal follicles	↑ AMH, E2 ↓ FSH	↑ FOXL2, CYP19A1

Grady *et al.* (2019)	Mare	BM	Mares (20–29 years)	Intraovarian injection (1 × 10^6^ cells)	14 and 19 weeks	=follicle count and oocyte recovery	=AMH	

Guo *et al.* (2013)	Murine	BM	Rats (9 months)	Intravenous injection (1–2 × 10^6^ cells)	12 weeks			↓ apoptosis

Huang *et al.* (2020)	Human	AF	Mice (12–14 months)	Intraovarian injection of cells	4 weeks	↑ follicle count	↑ AMH, E2 ↓ FSH ↑ FSHR, CYP19A1	↓ apoptosis ↑ PI3K/Akt activation (FOXL2) ↑genes for oocyte maturation (MSH4, GDF9, BMP15) ↑ DNA repair mechanisms

Kim *et al.* (2020)	Human	P	Rats (13–14 months)	Repeated (3×) intravenous injections (50 × 10^5^ cells)	Up to 5 weeks	↑ follicle growth	↑ AMH, E2	

Li *et al.* (2017)	Human	UC	Rats (12–14 months)	Intravenous injection (1 × 10^6^ cells)	Up to 4 weeks	↑ follicle count	↑ AMH, E2 ↓ FSH	↑ VEGF, HGF, IGF-1

Liu *et al.* (2021)	Human	AF	Mice (8 months) and RT (4 Gy) treatment	Repeated (3×) intravenous injections (5 × 10^6^; 7.5 × 10^6^ or 1 × 10^7^ cells)	1 and 2 weeks	↑ follicle count ↑ blastocyst formation rates	↑ AMH, E2 ↓ FSH	↓ apoptosis ↑ SOD2 ↑ Pi3K/Akt pathway activation (pFOXO3a)

Malard *et al.* (2020)	Bovine	AT	Bovines with infertile phenotype	Intraovarian injections (2.5 × 10^6^ cells)	3 weeks	↑ oocyte viability ↑ number of embryos		=gene expression for embryo quality (KTR8, PLAC8, SLC2A1, CASP3, PROX3, DOS2)

Pan *et al.* (2021)	Murine	UC	Mice (18 months)	Daily iIntravenous injections (1 × 10^7^ cells) for 3 weeks	4 weeks	↑ normal follicles	↑ AMH, E2, inhibin B =FSH	↓ apoptosis ↓ autophagy ↓ aging-related proteins (p53, p16, SOD1)

Tian *et al.* (2021)	Macaque	UC	Macaques (22–26 years)	Repeated (3×) intravenous injections (1 × 10^7^ cells)	8 months	↑ follicle count	↑ E2, testosterone, progesterone =FSH, LH, AMH	↓ apoptosis ↓ aging-related gene expression ↑ vessel density ↓ fibrosis

Wang *et al.* (2022)	Murine	BM	Mice (4–10 months)	Intravenous, intraperitoneal or intraovarian injection (6 × 10^5^ cells)	Up to 8 weeks	↑ follicle count ↑ oocyte number ↑ embryo formation	↑ E2 ↓ FSH, LH	↑mitochondrial biogenesis in oocytes
Yang *et al.* (2020b)	Human	UC	Mice (10 months)	Repeated intrabursal injection of exosomes for 3 weeks	3 weeks	↑ follicle growth ↑ normal oocytes ↑ litter number		↑ PI3K/Akt pathway activation (pAKt, pmTOR, FOXO3)

Zhang *et al.* (2022a)	Rodent	Men	Mice (10 months)	Periovarian deposition of alginate loaded with either 1 × 10^6^ cells or cell-derived mitochondria)	1 week	=follicle count		↑ gene expression of mitochondria- related pathways

AF, amniotic fluid; AMH, anti-Müllerian hormone; AT, adipose tissue; BM, bone marrow; BMP15, bone morphogenic protein 15; CYP19A1, cytochrome P450 19A1; E2, estradiol; FOXL2, forkhead box L2; GDF9, growth differentiation factor 9; HGF, hepatic growth factor; IGF1, insulin growth factor 1; mTOR, mammalian target of rapamycin; MSH4, MutS protein homolog 4; P, placenta; pAkt, phosphorylated Akt; PI3K/Akt, phosphatidylinositol 3-kinase/protein kinase B; pFOXO3a, phospho-forkhead box 03a; RT, radiotherapy; SOD, superoxide dismutase; UC, umbilical cord; VEGF, vascular endothelial growth factor.

With regard to gonadotoxic damage, 65 studies were selected. They were all aiming to characterize the effect of MSC use to reverse gonadotoxic injury in a murine model, except one study that used rabbits (Table 4). In 36 studies, cyclophosphamide was administered at different doses ranging from 50 to 200 mg/kg/day for just 1 or up to 15 days (Fu *et al.*, 2008, 2017; Deng *et al.*, 2021; Abd-Allah *et al.*, 2013; Lai *et al.*, 2013, 2014; Sun *et al.*, 2013; Takehara *et al.*, 2013; Kilic *et al.*, 2014; Liu *et al.*, 2014b; Xiao *et al.*, 2014; Zhu *et al.*, 2015; Song *et al.*, 2016; Badawy *et al.*, 2017; Ding *et al.*, 2017, 2020; Ling *et al.*, 2017, 2019, 2022a,b; Pan *et al.*, 2017; Bao *et al.*, 2018; Chen *et al.*, 2018, 2023; Herraiz *et al.*, 2018a; Huang *et al.*, 2018; Mohamed *et al.*, 2018; Besikcioglu *et al.*, 2019; Yang *et al.*, 2019a,b, 2020a; Zarbakhsh *et al.*, 2019; Zheng *et al.*, 2019; Feng *et al.*, 2020; Luo *et al.*, 2020; Shen *et al.*, 2020; Buigues *et al.*, 2021a; Çil and Mete, 2021; Jalalie *et al.*, 2021; Li *et al.*, 2021; Lv *et al.*, 2021; Park *et al.*, 2021, 2023; Salvatore *et al.*, 2021; Sen Halicioglu *et al.*, 2022; Geng *et al.*, 2022; Zhang *et al.*, 2022b, 2023; Cao *et al.*, 2023; Eslami *et al.*, 2023). In 17 studies, busulfan was used at doses ranging from 12 to 30 mg/kg/day for single or repeated administration (Table 4). In four studies (Ding *et al.*, 2017; Herraiz *et al.*, 2018a; Salvatore *et al.*, 2021; Buigues *et al.*, 2021a), different doses were selected and compared in order to mimic a model of mild and severe ovarian injury. Eight studies used cisplatin at doses from 2 to 50 mg/kg/day to induce an ovarian damage model (Liu *et al.*, 2014a; Wang *et al.*, 2017; Sun *et al.*, 2019; Cui *et al.*, 2020; Hong *et al.*, 2020; Zhang *et al.*, 2021a; Luo *et al.*, 2022; Qu *et al.*, 2022), while others employed whole body irradiation of 3.2 and 4 Gy, respectively (El-Derany *et al.*, 2021; Liu *et al.*, 2021), paclitaxel (Elfayomy *et al.*, 2016), epirubicin (Guo *et al.*, 2019), vinylcyclohexene diepoxide (Zhang *et al.*, 2021b; Jiao *et al.*, 2022), hydrogen peroxide (Liu *et al.*, 2019), and zona pellucida 3 peptide (Li *et al.*, 2019; Zhang *et al.*, 2021c) to repair ovarian damage. Using MSCs encounters the same great variability in terms of stem cell sources, concentrations and modes of administration as do models of natural aging and rejuvenation. Moreover, in only 47 of the 65 studies (72%) was there a known time frame between use of gonadotoxic agents and MSC treatment, ranging from 0 to 24 h in 21 studies, up to 1 week in 20 studies, and much longer (from 10 days to 6 weeks) in the remaining six studies. This is a crucial factor for the ovarian damage model, since some events, like DNA damage and apoptosis induction, arise just hours after drug exposure, while tissue remodeling and fibrosis occur over subsequent weeks. Such variability makes it difficult to evaluate the actual healing properties and effects of MSCs on ovarian reserve injury, and may limit the applicability of the results in a clinical setting.

**Table 4:** Effects of mesenchymal stem cells in ovarian damage models.

Author	Mesenchymal stem cells		Experimental model					Follicle and oocyte outcomes	Hormone activity	Other biological effects
	Stem cell origin	Stem cell source	Animal model	CHT/RT	Interval between CHT/RT and stem cell treatment	Stem cell use	Time- points			
Abd-Allah *et al.* (2013)	Rabbit	BM	CHT- treated rabbits	Cy 50 mg/kg followed by Cy 8 mg/kg/day for 14 days	0	Intravenous injection (5 × 10^6^ cells)	6 weeks	↑ follicle count ↑ follicle growth	↑ E2, AMH ↓ FSH	↓ apoptosis ↑ VEGF; ↓ fibrosis

Badawy *et al.* (2017)	Murine	BM	CHT-treated mice	Cy 80 mg/kg	4 days	Intravenous injection (5 × 10^5^ cells)	3 weeks	↑ follicle count	↑ E2 ↓ FSH	

Bao *et al.* (2018)	Murine	BM	CHT-treated mice	Cy 50 mg/kg followed by 8 mg/kg/day for 14 days	1 week	Intraovarian injection (1 × 10^6^ cells)	4 weeks	↑ follicle count	↑ E2 ↓ FSH	↓ apoptosis ↑ proliferation gene expression

Besikcioglu *et al.* (2019)	Human	UC	CHT-treated rats	Intraperitoneal Cy 200 mg/kg injections (day 1 and 8)	0	Repeated (2×) intraperitoneal injections (2 × 10^6^ cells)	1 day after last injection	↑ follicle count	similar AMH	

Buigues *et al.* (2021a)	Human	BM	CHT-treated mice	Mild: Cy 12 mg/kg + busulfan 1,2 mg/kg; Severe: Cy 120 mg/kg + busulfan 12 mg/kg for 14 days	1 week	Repeated injections of GCS-F for 2 weeks for stem cell mobilization	2 weeks	↑ follicle count ↑ follicle growth ↑ pregnancy rates and number of pups		↑ proliferation and ↓ apoptosis pathways (proteomic study)

Cao *et al.* (2023)	Human	S (iPSCs)	CHT-treated mice	Intravenous Cy 120 mg/kg injections (day 1 day 8)	0	Repeated intravenous injections every 3 days for 2 weeks (200 µg EVs)	18 days	↑ primordial follicles		↓ apoptosis ↑ PI3K/Akt activation

Chen *et al.* (2018)	Murine	BM	CHT-treated rats	Cy 50 mg/kg followed by 8 mg/kg/day for 14 days	NA	Intraovarian injection (2 × 10 6 cells +/− heat-shock pretreatment)	Up to 60 days	↑ follicle count	↑ E2 ↓ FSH	↓ apoptosis

Chen *et al.* (2023)	Human	UC	CHT-treated rats	CY 83.5 mg/kg and busulfan 20.8 mg/kg	1 day	Intraovarian injection (5 × 10^6^ cells +/−expressing human HGF)	4 weeks	↑ follicle count ↑ follicle growth ↓ atretic follicles	↑ AMH, E2 ↓ FSH	↓ fibrosis (collagen deposition) ↑ HGF, VEGF ↑ revascularization (CD34)

Çil and Mete (2021)	Murine	AT	CHT-treated mice	Cy 50 mg/kg followed by 8 mg/kg/day for 13 days	NA	Intraovarian injection (5 × 10^4^ cells)	8 weeks	↑ early-stage follicles ↓ atretic follicles		↑ PI3K/Akt activation in early-stage follicles (mTOR)

Cui *et al.* (2020)	Human	UC	CHT-treated rats	Cisplatin 2 mg/kg/day for 7 days	1 week	Intravenous injection (2 × 10^6^ cells)	1 week	↑ pregnancy rates	↑ E2 ↓ FSH, LH	↓ fibrosis

Deng *et al.* (2021)	Human	UC	CHT-treated mice	Cy 120 mg/kg + busulfan 30 mg/kg	1 week	Repeated (2×) injections (1 × 10^6^ cells) every 48 hours	2 weeks	↑ early-stage follicles;	↑ E2 ↓ FSH	↓ apoptosis ↑ Pi3K/Akt pathway activation (Pakt) ↑ Il10, TSG6 and VEGF ↓IL6 and IlB1
Ding *et al.* (2017)	Human	AF	CHT-treated mice	Mild: Cy 70 mg/kg, for 2 weeks; Moderate: (120 mg/kg for 1 week, 70 mg/kg for 1 week; Severe: 120 mg/kg 2 for weeks	NA	Intraovarian injection of cells	4 weeks	↑ follicle count ↑ number of pups	↑ AMH, E2 ↓ FSH	↑ folliculogenesis markers (KI67, AMH, FSHR)

Ding *et al.* (2020)	Human	UC	CHT-treated mice	Cy 120 mg/kg	NA	Exosome injection (10^12^ particles/ml +/− anti-miR-17-5p)	4 weeks	↑ follicle count ↑ follicle growth	↑ AMH, E2 ↓ FSH	↓ apoptosis

Elfayomy *et al.* (2016)	Human	UC	CHT-treated rats	Paclitaxel 7.5 mg/kg	1 week	Intraovarian injection (2 × 10^6^ cells)	Up to 6 weeks	↑ follicle count ↑ antral follicles ↑ normal follicles	↑ E2 ↓ FSH	↑ TGFβ

El-Derany *et al.* (2021)	Murine	BM	RT-treated mice	Whole body irradiation (3.2 Gy)	1 week	Intravenous injection (2 × 10^6^ cells)	4 weeks	↑ follicle count	↑ E2 ↓ FSH	↓ apoptosis ↑ follicle activation (FOXO1-3, GDF9, TEAD, YAP1) ↓ TGF-β; ↑ β-catenin

Eslami et al. (2023)	Murine	BM	CHT-treated mice	Cy for 15 days	1 day	Intravenous injection (cells or extracellular vesicles)	4 and 8 weeks	↑ follicle count ↑ number of pups	↑ AMH, E2, FSHR ↓ FSH	↑ GDF9

Feng *et al.* (2020)	Human	AF	CHT-treated rats	Cy 83.5 mg/kg and busulfan 20.8 mg/kg	1 day	Intravenous or intraovarian injection (4 × 10^6^ cells)	4 weeks	↑ follicle count	↑ AMH, E2 ↓ FSH	↑ proliferation markers (↑ JNK2, =p38 and serpin E1)

Fu *et al.* (2008)	Murine	BM	CHT-treated rats	Cy 50 mg/kg followed by Cy 8 mg/kg/day for 14 days	NA	Intraovarian injection (2 × 10^6^ cells)	Up to 8 weeks	↑ follicle count =follicle growth	↑ E2 ↓ FSH	↓ apoptosis

Fu *et al.* (2017)	Murine	BM	CHT-treated rats	Cy 50 mg/kg followed by Cy 8 mg/kg/day for 14 days	0	Peritoneal injection (1 × 10^9^ cells lentivirus-miR-21 transfected cells)	Up to 8 weeks	↑ follicle count	↑ AMH, E2 ↓ FSH	↓ apoptosis

Geng *et al.* (2022)	Human	AF	CHT-treated mice	Cy 70 mg/kg/day for 7 days followed by Cy 30 mg/kg/every 2 days for 14 days	NA	Intravenous injection every 2 days for 4 weeks (exosomes)	NA	↑ follicle count		↓ apoptosis

Guo *et al.* (2019)	Human	Men	CHT-treated mice	Epirubicin 0.01 mg/kg for 7 days	1 week	Intravenous injection (1 × 10^6^ cells +/− 3-day BSTCR gavage)	4 weeks	↑ follicle count ↓ follicle atresia	↑ AMH, E2 ↓ FSH	↓ apoptosis
Herraiz *et al.* (2018a)	Human	BM	CHT-treated mice	Mild: Cy 12 mg/kg + busulfan 1,2 mg/kg; Severe: Cy 120 mg/kg + busulfan 12 mg/kg for 14 days	1 week	Intravenous injection (1 × 10^6^ cells) after GCS-F treatment	2 weeks	↑ follicle count ↑ follicle growth ↑ fertilization rates ↑ pregnancy rates and number of pups		↓ apoptosis

Hong *et al.* (2020)	Human	UC	CHT-treated mice	Cisplatin 50 mg/kg	0	Daily intraperitoneal injection for 5 days (30–50 µl conditioned medium)	8 weeks	↑ follicle count	↑ AMH	↓ apoptosis ↑ follicle activation (PI3K/Akt)

Huang *et al.* (2018)	Human	AT	CHT-treated mice	Cy 120 mg/kg	NA	Repeated injections of exosomes	4 weeks	↑ follicle count ↑ follicle growth	↑ AMH, E2 ↓ FSH	↓ apoptosis ↑proliferation markers (SMAD 2,3,5)

Jalalie *et al.* (2021)	Human	UC	CHT-treated mice	Cy 50 mg/kg for 15 days	1 day	Intravenous injection (1 × 10^6^ cells)	1 week	↑ follicle count ↑normal oocytes	↑ E2	↓ apoptosis ↓ fibrosis

Jiao *et al.* (2022)	Human	UC	CHT-treated mice	VCD 80 mg/kg/day for 10 days	NA	Injection (1 × 10^5^ cells)	2 weeks, 4–6 months	↑ follicle count	↑ E2 ↓ FSH	↓ apoptosis ↑ growth factor secretion (VEGF, EGF, SCF)

Kilic *et al.* (2014)	Murine	BM	CHT-treated rats	Cy 200 mg/kg twice	1 day	Repeated (4×) injections (1 × 10^6^ cells +/− GnRH agonists) for 2 weeks	1 days after last injection	↑ follicle count		↓ apoptosis

Lai *et al.* (2013)	Human	AF	CHT-treated mice	Cy 120 mg/kg + busulfan 30 mg/kg	1 week	Intraovarian injection (2–5 × 10^3^ cells)	8 weeks	↑ follicle count ↑ oocyte number	↑ AMH	↑ FSHR

Lai *et al.* (2014)	Murine	S	CHT-treated mice	Cy 120 mg/kg + busulfan 30 mg/kg	1 week	Intravenous injection (2 × 10^6^ cells)	up to 8 weeks	↑ follicle count ↑ pregnancy rates		↑ AMH expression ↓ proinflammatory cytokines levels

Li *et al.* (2019)	Human	P	Chemical-treated mice	pZP3 0.15 ml for 14 days	NA	Tail vein injection (1 × 10^6^ cells)	2 weeks	↑ follicle growth ↓ follicle atresia	↑ E2 ↓ FSH, LH	↓ apoptosis ↓ endoplasmic reticulum stress markers (IRE1α, XBP1, GRP78)

Li *et al.* (2021)	Human	UC	CHT-treated mice	Cy 120 mg/kg/week for 2 weeks	2 weeks	Repeated (2×) intraperitoneal injections (exosomes) for 2 weeks	8 weeks	↑ follicle count ↑ follicle growth	↑ E2, AMH, FSHR ↓ FSH	↓ apoptosis

Ling *et al.* (2017)	Human	AT	CHT-treated rats	Cy 50 mg/kg followed by Cy 8 mg/kg/day for 14 days	1 day	Intravenous injection (4 × 10^6^ cells +/− LIPUS pretreatment)	4 and 8 weeks	↑ follicle count	↑ AMH, E2 ↓ FSH	↓ apoptosis ↓ proinflammatory cytokines ↑ VEGF
Ling *et al.* (2019)	Human	AF	CHT-treated rats	Cy 50 mg/kg followed by Cy 8 mg/kg/day for 14 days	1 day	Intraovarian injection (4 × 10^6^ cells)	2,4 and 8 weeks	↑ follicle growth ↓ follicle atresia	↑ E2 ↓ FSH, LH	↓ apoptosis ↑ growth factor production (GCS-F, FGF2, IGF1, HGF, VEGF)

Ling *et al.* (2022a)	Human	P	CHT-treated rats	Cy 50 mg/kg followed by Cy 8 mg/kg/day for 14 days	1 day	Intravenous injection (4 × 10^6^ cells +/− LIPUS pretreatment)	1 day, 3 and 6 weeks	↑ follicle count ↑ number of pups		↑ SDF-1 for stem cell homing

Ling *et al.* (2022b)	Human	P	CHT-treated rats	Cy 50 mg/kg followed by Cy 8 mg/kg/day for 14 days	1 day	Intravenous injection (4 × 10^6^ cells +/− AMD3100 or +/−LY294002)	1 day, 3 and 6 weeks	↑ follicle count	↑ E2, AMH ↓ FSH	↓ apoptosis ↑neoangiogenesis (VEGF, VEGFR2)

Liu *et al.* (2014b)	Human	Men	CHT-treated mice	Cy 70 mg/kg	1 week	Intraovarian injection (1 × 10^4^ cells)	up to 3 weeks	↑ follicle count	↑ AMH, E2 ↓ FSH	↑ folliculogenesis markers (AMH, inhibin A, inhibin B, FSHR)

Liu *et al.* (2019)	Human	AF	Chemical-treated mice	Intraovarian 10% hydrogen peroxide injection (1 minute)	NA	Intraperitoneal injection (1 × 10^6^ cells)	1 and 2 weeks	↑ follicle count ↓ follicle atresia ↑ estrous cycle recovery =fertility rates	↑ E2 ↓ FSH	↑ follicle activation (FOXL2, OCT4, GDF9, SCF, LIF)

Luo *et al.* (2020)	Human	P	CHT-treated mice	Cy 120 mg/kg + busulfan 30 mg/kg	2 weeks	Intravenous injection (1 × 10^6^ cells)	1 week	↑ normal follicles	↑ E2 ↓ FSH	↓ apoptosis; ↑ FSHR

Luo *et al.* (2022)	Human	UC	CHT- treated rats	Cisplatin 2 mg/kg/day for 7 days	NA	Intravenous injection (1 × 10^6^ cells)	1 week	↑follicle count	↑ E2, Tst ↓ FSH, LH	↑ CYp17A1 expression ↓ apoptosis of Theca cells

Lv *et al.* (2021)	Human	UC	CHT-treated mice	Cy 120 mg/kg + busulfan 30 mg/kg	1 week	Repeated (3×) intravenous injections (2 × 10^6^ cells) for 3 weeks	Up to 8 weeks	↑follicle count ↑ follicle growth ↑ litter number	↑ AMH, E2, inhibin A and B, FSHR	

Mohamed et al. (2018)	Human	BM	CHT-treated mice	Cy 70 mg/kg + busulfan 12 mg/kg	1 week	Intraovarian injection (5 × 10^5^ cells)	Up to 8 weeks	↑ follicle count	↑ E2, AMH ↓ FSH	↑ folliculogenesis markers (FSHR, inhibin A, inhibin B, AMH)

Mohamed *et al.* (2019)	Human	UC	CHT-treated mice	Cy 70 mg/kg + busulfan 12 mg/kg	1 week	Intraovarian injection (5 × 10^5^ cells)	Up to 8 weeks	↑ follicle count; ↑ pregnancy rates and number of pups	↑ E2, AMH ↓ FSH	

Pan *et al.* (2017)	Human	UC or AT	CHT-treated mice	Cy 80 mg/kg/day for 14 days	NA	Intraovarian injection (1 × 10^6^ cells)	3 months	↑ follicle count	↑ E2, AMH ↓ FSH, LH	
Park *et al.* (2021)	Human	BM	CHT-treated mice	Cy 120 mg/kg + busulfan 30 mg/kg	1 day	Injection (5 × 10^5^ cells)	2 weeks	↑ follicle count ↑ follicle growth	↑ E2	

Park *et al.* (2023)	Human	BM/UC	CHt-treated mice	Cy 120 mg/kg + busulfan 30 mg/kg	1 week	Intravenous injection (1 × 10^5^ cells or 1 × 10^7^ exosomes)	2 weeks	↑ follicle count ↑ pregnancy rates ↑ number of pups	↑ E2, AMH ↓ FSH	↓ fibrosis (collagen deposition)

Qu *et al.* (2022)	Human	UC	CHT-treated rats	Cisplating 1 mg/kg/day for 14 days	0	Intravenous injection (exosomes)	1,2,3 and 4 weeks		↑ E2, AMH ↓ FSH	↓ apoptosis; ↑ neoangiogenesis (CD31 and VEGF)

Salvatore *et al.* (2021)	Human	AT	CHT-treated mice	Mild: Cy 120 mg/kg + busulfan 12 mg/kg; Severe: Cy 120 mg/kg + busulfan 30 mg/kg	1 week	Intravenous injection (1 × 10^6^ cells)	4 weeks	↑ follicle count =oocyte retrieval ↑ fertilization rates		

Sen Halicioglu et al. (2022)	Rodent	AT	CHT-treated rats	Cy 120 mg/kg/week for 2 weeks	1 week	Intraperitoneal injection (1 × 10^6^ cells)	2 weeks	↑ follicle count	↑ AMH	↓ apoptosis ↑ connexin 43 ↓ pannexin 1

Shen *et al.* (2020)	Human	UC	CHT-treated mice	Cy 50 mg/kg followed by Cy 8 mg/kg (14 days)	10 days	Repeated (4×) Intravenous injections (1 × 10^6^ cells) for 4 weeks	9 weeks	↑ follicle count ↓ follicle atresia =estrous cycle recovery	↑ AMH, E2	

Song *et al.* (2016)	Human	UC	CHT-treated mice	Cy 200 mg/kg followed by Cy 8 mg/kg/day for 15 days	2 weeks	Tail vein or intraovarian injection (1 × 10^6^ cells)	Up to 3 weeks	↑ follicle count ↑ secondary follicles	↑ E2, AMH ↓ FSH	↓ apoptosis

Sun *et al.* (2019)	Murine	BM	CHT-treated mice	5 mg/kg cisplatin	0	Repeated (3×) intraperitoneal injections (exosomes)	2 weeks	↑ follicle count		↓ apoptosis

Takehara *et al.* (2013)	Murine	BM	CHT-treated rats	Cy 50 mg/kg followed by Cy 8 mg/kg/day for 13 days	1 day	Intraovarian injection (5 × 10^6^ or 1 × 10^7^ cells)	8 weeks	↑ follicle count ↑ litter numbers		↑ neoangiogenesis (CD34) ↑ growth factor secretion (VEGF, IGF-1, HGF)

Wang *et al.* (2017)	Human	Men	CHT-treated mice	Cisplatin 2 mg/kg/day for 7 days	NA	Repeated (2×) tail vein injections (1 × 10^7^ cells)	1 and 3 weeks	↑ follicle count	↑ E2 ↓ FSH	↓ apoptosis

Xiao *et al.* (2014)	Murine	AF	CHT-treated rats	Cy 200 mg/kg + busulfan 20 mg/kg	6 weeks	Intraovarian injection (5 × 10^5^ cells)	Up to 8 weeks	↑ normal follicles ↑ litter number		

Yang *et al.* (2019a)	Human	UC	CHT-treated mice	CY 200 mg/kg and busulfan 20 mg/kg	2 weeks	Repeated tail vein injection of exosomes for 1 week	6 weeks	↑ follicle count	↑ E2 ↓ FSH	↑vascularization ↑ growth factor secretion (VEGF, IGF-1)

Yang *et al.* (2019b)	Human	UC	CHT-treated mice	Cy 40 mg/kg/day for 15 days	NA	Intraovarian injection (2 × 10^5^ cells +/− collagen scaffold)	4 weeks	↑ follicle count	↑ E2, AMH ↓ FSH	↑ vascularization
Yang *et al.* (2020a)	Murine	BM	CHT-treated rats	Cy 50 mg/kg followed by 8 mg/kg for 14 days	NA	Repeated intraperitoneal injections (1 × 10^6^ cells or exosomes) for 2 weeks	4 weeks	↑ follicle count ↑ normal follicles	↑ E2, AMH	↓ apoptosis ↑ PI3K/Akt pathway activation

Zarbakhsh *et al.* (2019)	Murine	BM	CHT-treated rats	Cy 50 mg/kg followed by 8 mg/kg for 13 days	NA	Intraovarian injection (2 × 10^6^ cells +/− carnitine)	4 weeks	↑ follicle count	↑ E2; ↓ FSH	↓ apoptosis (Bcl2/Bax)

Zhang *et al.* (2021a)	Human	UC	CHT- treated rats	Cisplatin 2.5 mg/kg/day for 7 days	NA	Conditioned medium encapsulated inside ORP grafted intraperitoneally	3 weeks	↑ follicle count ↑ follicle growth ↑ pregnancy rates	↑ E2, AMH ↓ FSH	

Zhang *et al.* (2021b)	Human	Men	CHT- treated rats	VCD 80 mg/kg/day for 15 days	NA	Repeated (4×) Intraovarian injections) (5 × 10^5^ cells or exosomes) for 28 days	4 weeks	=follicle density ↑ litter number	↑ E2, AMH ↓ FSH	↓ apoptosis ↓ collagen I, ↑collagen IV, fibronectin 1 and laminin

Zhang *et al.* (2021c)	Human	UC	Chemical-treated rats	pZP3 400 µl injection	0	Intraovarian injection (5 × 10^5^ cells)	3 weeks	↑ follicle count	↑ E2 ↓ FSH, LH	

Zhang *et al.* (2022a)	Human	UC	CHT-treated mice	Cy 120 mg/kg + busulfan 30 mg/kg	1 week	Intravenous injection (1 × 10^6^ cells)	4 weeks	↑ follicle count	↑ E2, AMH ↓ FSH	↓ apoptosis

Zhang *et al.* (2023)	Human	BM	CHT-treated mice	Cy 50 mg/kg for 14 days	5 days	Intraovarian injection (exosomes)	10 weeks	↑ follicle count	↑ E2, AMH ↓ FSH	↓ apoptosis

Zheng *et al.* (2019)	Human	UC	CHT-treated rats	Cy 50 mg/kg followed by 8 mg/kg/day for 14 days	0	Intravenous injection (5 × 10^6^ cells)	2 and 4 weeks	↑ follicle growth	↑ E2, AMH ↓ FSH	↓ apoptosis ↑ TrkA and NGF expression ↓ FSHR expression

Zhu *et al.* (2015)	Human	UC	CHT-treated rats	Cy 50 mg/kg followed by 8 mg/kg/day for 14 days	NA	Intravenous and intraovarian injection (1 × 10^6^ cells)	Up to 6 weeks	↑ secondary follicles ↑ pregnancy rates	↑ E2 ↓ FSH	

AF, amniotic fluid; AMH, anti-Müllerian hormone; Angpt1, angiopoietin 1; AT, adipose tissue; BAX, BCL2-associated X; Bcl2, BCL2, B-cell lymphoma 2; BM, bone marrow; CCHC domain-containing protein 11Bpil3; CHT, chemotherapy; cxcr4, C-X-C Motif Chemokine Receptor 4; Cy, cyclophosphamide; E2, estradiol; EGF, epithelial growth factor; FGF2, fibroblastic growth factor 2; FOXL2, forkhead box L2; FOXO1-3, forkhead box 1-3; GCS-F, granulocyte colony-stimulating factor; GDF9, growth differentiation factor 9; GRP78, Glucose-Regulated Protein 78; HGF, hepatic growth factor; IGF1, insulin growth factor 1; IRE1α, Inositol-requiring enzyme 1 α; JNK2, JNK [c-Jun N-terminal kinase] isoform 2; LIF, leukemia inhibiting factor; LIPUS, low-intensity pulsed ultrasound; Men, menstrual blood; NGF, nerve growth factor; Onecut2, OCT4, octamer-binding transcription factor 4; One Cut Homeobox 2; ORP, ovarian reproductive patch; P, placenta; PI3K/Akt, phosphatidylinositol 3-kinase/protein kinase B; pZP3, zona pellucida 3 peptide; RT, radiotherapy; S, skin; SCF, stem cell factor; TEAD, transcriptional enhanced associate domain; TGFβ, transforming growth factor β; Trk1, potassium transport protein 1; TSG6, tumor necrosis factor-(TNF) stimulated gene-6; UC, umbilical cord; VCD, vinylcyclohexene diepoxide; VEGF, vascular endothelial growth factor; XPB1, X-Box Binding Protein 1; YAP1, yes-associated protein 1; Zcchc11, zinc-finger.

#### Ovarian rejuvenation and gonadotoxic damage healing: outcomes and mechanism of action

Outcomes for both ovarian rejuvenation and gonadotoxic damage healing were relatively homogeneous, both showing a positive impact of MSCs on follicle count through regulation of a number of key processes in the ovarian microenvironment. A decrease in apoptosis in all ovarian compartments, including GCs, theca cells and ovarian stroma, was evidenced in the vast majority of the studies. MSCs appear to significantly downregulate various signals responsible for triggering the apoptosis cascade, involving upregulation of antiapoptotic molecules such as B-cell lymphoma 2 (BCL2), survivin (Huang *et al.*, 2020), and NR4A1, which look to be specific to theca cells (Luo *et al.*, 2022), and a shift in the relation between pro- and antiapoptotic signals; for example, the BCL2/BCL2-associated X (BAX) ratio (Zarbakhsh *et al.*, 2019; Geng *et al.*, 2022; Ling *et al.*, 2022b; Luo *et al.*, 2022; Qu *et al.*, 2022; Zhang *et al.*, 2023). This is probably a result of the paracrine signaling of MSCs, which are rich in growth factors like VEGF, HGF, and IGF-1, and able to promote cell survival and proliferation *in vitro* and *in vivo* (Uzumcu *et al.*, 2006; Polonio *et al.*, 2020; Chen *et al.*, 2023).

MSC paracrine signaling has been shown to target different growth factors, including VEGF, HGF, TGFβ, IGF-1, and nerve growth factor (NGF), whose presence was first confirmed in MSC *in vitro*, and then detected in the ovaries after *in vivo* administration of MSCs (Abd-Allah *et al.*, 2013; Sun *et al.*, 2013; Takehara *et al.*, 2013; Elfayomy *et al.*, 2016; Ling *et al.*, 2017, 2019, 2022b; Zheng *et al.*, 2019; Yang *et al.*, 2019a; Deng *et al.*, 2021; Jiao *et al.*, 2022). Elevated proangiogenic growth factor levels may enhance tissue revascularization, whose increase is sustained by development of theca cells around secondary follicles and is essential for their further growth (Takehara *et al.*, 2013; Yang *et al.*, 2019a,b; Tian *et al.*, 2021; Buigues *et al.*, 2021a; Chen *et al.*, 2023). On the other hand, proinflammatory cytokines Il6 and Ilβ1 were found at decreased levels after MSCs use (Lai *et al.*, 2014; Ling *et al.*, 2017; Deng *et al.*, 2021). Such regulation of the cytokine environment, favoring neovascularization (Qu *et al.*, 2022), and control of inflammation, may be why collagen deposition leading to fibrosis is also reduced (Abd-Allah *et al.*, 2013; Jalalie *et al.*, 2021; Tian *et al.*, 2021; Zhang *et al.*, 2021b; Chen *et al.*, 2023; Park *et al.*, 2023).

This favorable paracrine signaling results in not only GC survival, but also broader beneficial effects on the ovarian reserve. Indeed, hormone function appeared to be restored, showing improved hormone values in many studies (decrease in FSH and increase in AMH and E2). Along with endocrine function restoration, effective protection of the follicle pool was also evidenced by greater numbers of oocytes and embryos (Lai *et al.*, 2013; Sun *et al.*, 2013; Herraiz *et al.*, 2018a; Grady *et al.*, 2019; Liu *et al.*, 2019; Malard *et al.*, 2020; Jalalie *et al.*, 2021; Liu *et al.*, 2021; Salvatore *et al.*, 2021; Wang *et al.*, 2022), higher pregnancy rates, and larger litters (Takehara *et al.*, 2013; Lai *et al.*, 2014; Xiao *et al.*, 2014; Zhu *et al.*, 2015; Ding *et al.*, 2017; Liu *et al.*, 2019; Herraiz *et al.*, 2018a; Mohamed *et al.*, 2019; Cui *et al.*, 2020; Yang *et al.*, 2020b; Buigues *et al.*, 2021a; Lv *et al.*, 2021; Zhang *et al.*, 2021a,b; Ling *et al.*, 2022a; Eslami *et al.*, 2023; Park *et al.*, 2023).

In terms of follicle quality, enhanced follicle growth and improved follicle morphology were often observed. A number of potential mechanisms could be involved in this observation, since follicle growth and function are regulated by a complex interaction of pathways. This includes the ability of follicles to remain quiescent in the ovarian cortex, with oocytes in meiotic arrest, and at the same time ready for recruitment and further growth (Grosbois *et al.*, 2020). One of the main biological functions impacted by the MSC secretome does appear to be primordial follicle activation. Interaction with the PI3K/Akt pathway was indeed demonstrated in several studies, resulting in better follicle growth (Ding *et al.*, 2018; Liu *et al.*, 2019, 2021; Hong *et al.*, 2020; Huang *et al.*, 2020; Yang *et al.*, 2020a,b; Çil and Mete, 2021; Deng *et al.*, 2021; El-Derany *et al.*, 2021; Cao *et al.*, 2023). Other cell proliferation signals, like SMADs and c-Jun N-terminal kinase (JNK2) (Bao *et al.*, 2018; Huang *et al.*, 2018; Feng *et al.*, 2020), were also found, as were cell-cell interaction signals such as connexin 43 expression (Sen Halicioglu *et al.*, 2022). Rapid oocyte growth and meiotic resumption require an efficient engine to ensure complete maturation and good quality embryos. This is facilitated by the presence of a large mitochondrial mass and abundance of substrates, including glucose and fatty acids for oxidative phosphorylation (Al-Zubaidi *et al.*, 2021). The impact of MSCs on mitochondrial function in oocytes was also explored in one study, which found a more substantial relevant increase in mitochondrial DNA in the presence of MSCs, a key step allowing further meiotic resumption (Wang *et al.*, 2022).

The effect on mitochondria is part of a more extensive influence that MSCs have on cell function, which has been shown by several authors to exert anti-aging properties (Cacciottola *et al.*, 2021a). Mitochondrial function and control of oxidative stress in cells are among critical factors in cell senescence, and their regulation may explain the rejuvenating effect of stem cell therapy. Moreover, MSCs were found to reverse other specific signaling pathways associated with aging, including upregulation of DNA damage repair mechanisms, namely phosphorylated histone H2AX (γH2AX), breast cancer 1 (BRCA1), poly [ADP-ribose] polymerase 1 (PARP1), and X-ray repair cross complementing 6 (XRCC6) (Huang *et al.*, 2020), as well as cell cycle progression (El-Derany *et al.*, 2021; Tian *et al.*, 2021). One study explored the possibility of enhancing ovarian tissue quality by injecting MSC-derived mitochondria into the periovarian space, exhibiting upregulation of gene pathways related to mitochondrial function and energy supply (Zhang *et al.*, 2022a). The experiment did not, however, demonstrate any significant effect on the ovarian reserve, confirming that natural aging is more challenging to reverse than iatrogenic treatments.

Poor follicle quality may also be explained by other less explored mechanisms. Endoplasmic reticulum (ER) stress was investigated in one study as a potential trigger of follicle death, using markers like inositol-requiring enzyme 1α and glucose-regulated protein 78 (Li *et al*, 2019). MSCs appeared to reverse this cell dysfunction, resulting in restored GC survival. ER stress is caused by the accumulation of unfolded or misfolded proteins caused by various pathological conditions, including oxidative stress and inflammation. It leads to organelle swelling in the form of cytoplasmic vacuoles, which may trigger follicle death by atresia (Hay *et al.*, 1976). While massive ER dysfunction owing to complete loss of calcium homeostasis and membrane lipid damage is very hard to reverse, moderate ER stress levels may allow oocyte maturation (Lin *et al.*, 2012; Takahashi *et al.*, 2019). It is therefore important to act upon this cell mechanism to ensure the development of normal and fertilizable oocytes, having completed maturation, especially after *in vitro* growth (Harada *et al.*, 2015; McLaughlin *et al.*, 2018).

### MSCs for ovarian tissue transplantation

Twelve studies on ovarian tissue transplantation were identified and included in the review (Table 5). The majority of studies on human ovarian tissue were published by two research groups in China (Xia *et al.*, 2015; Cheng *et al.*, 2022) and two research groups in Europe, namely the UCL Gynecology Research Unit in Brussels, Belgium (Manavella *et al.*, 2018, 2019; Cacciottola *et al.*, 2020, 2021b,c) and the Reproductive Medicine Research Group in Valencia, Spain (Herraiz *et al.*, 2018a; Buigues *et al.*, 2021a,b). Two studies involved autologous ovarian transplantation in murine models (Damous *et al.*, 2018; Yang *et al.*, 2020b), where MSCs were either injected into the ovaries or systemically, taking advantage of their homing capacities, or grafted locally with the help of biocompatible scaffolds (fibrin or Matrigel).

**Table 5:** Effects of mesenchymal stem cells in models of ovarian tissue transplantation.

Author	Mesenchymal stem cells		Experimental model			Follicle and oocyte outcomes	Other biological effects
	Stem cell origin	Stem cell source	Stem cell use	Animal model	Time-points		
Buigues *et al.* (2021a)	Human	BM and UC	Repeated intravenous injections of GSC-F for 2 weeks	Human OT xenograft to mice 1 week before cell injection	14 days	=follicle count ↑ follicle growth	↑ microvessel density ↑ angiogenesis, protein synthesis and autophagy (proteomic analysis)

Buigues *et al.* (2021b)	Human	BM	Intravenous injection of 1 × 10^6^, or 3 × 10^5^ CD133+ cells	Human OT xenograft to mice 1 week before cell injection	7, 14 days		↑ vascularization, proliferation ↓ apoptosis (transcriptomic analysis)

Cacciottola *et al.* (2020)	Human	AT	In fibrin scaffolds (1.5 × 10^6^ cells)	Human OT xenograft to mice 2 weeks after fibrin scaffold implant	24 weeks	=follicle density ↑ primordial follicles =antral follicles	=AMH and E2 ↓ AMHRII in growing follicles

Cacciottola *et al.* (2021b)	Human	AT	In fibrin scaffolds (1.5 × 10^6^ cells)	Human OT xenograft to mice 2 weeks after fibrin scaffold implant	3 and 10 days	↑ follicle density ↑ primordial follicles	↓ follicle apoptosis ↓ PI3K/Akt pathway activation =Hippo pathway disruption

Cacciottola *et al.* (2021c)	Human	AT	In fibrin scaffolds (1.5 × 10^6^ cells)	Human OT xenograft to mice 2 weeks after fibrin scaffold implant	3, 10 and 21 days		Earlier reperfusion ↓ hypoxia signaling ↑ VEGF =oxidative stress-related damage

Cheng *et al.* (2022)	Human	UC	In Matrigel scaffolds (1 × 106 +/− hypoxia-treated cells)	Human OT xenograft to mice	3,7 days	↑ follicle density ↑ primordial follicles	↓ follicle apoptosis ↑ vascularization ↑ AMH, E2 and Prog ↓ FSH

Damous *et al.* (2018)	Murine	AT	Conditioned medium	Autologous ovarian transplantation + intraovarian secretome injection	4–5 weeks	=estrous cycle resumption	↓ follicle apoptosis

Herraiz *et al.* (2018a)	Human	BM	Intravenous injection of 1 × 10^6^ cells, or 3 × 10^5^ CD133+ cells	Human OT xenograft to mice 1 week before cell injection	7 and 14 days	↑ follicle growth	↑ microvessel density

Manavella *et al.* (2018)	Human	AT	In fibrin scaffolds (1.5 × 10^6^ cells)	Human OT xenograft to mice 2 weeks after fibrin scaffold implant	7 days	↑ follicle density	Earlier reoxygenation ↑ revascularization

Manavella *et al.* (2019)	Human	AT	In fibrin scaffolds (1.5 × 10^6^ cells)	Human OT xenograft to mice 2 weeks after fibrin scaffold implant	7 days		↑ revascularization ↑ VEGF and bFGF

Yang *et al.* (2020b)	Human	UC	Exosomes	Newborn ovary autograft in mice	1 and 18 days	↑ follicle growth	↑ pAkt, mTOR and FOXO3

Xia *et al.* (2015)	Human	BM	In Matrigel scaffolds (5 × 10^5^ cells)	Human OT xenograft to mice	3, 7 and21 days	↑ primordial follicles	↓ follicle apoptosis ↑ microvessel density ↑graft perfusion

AMH, anti-Müllerian hormone; AMHRII, anti-Müllerian hormone receptor II; AT, adipose tissue; bFGF; basic fibroblastic growth factor; BM, bone marrow; E2, estradiol; FOX03, forkhead box 03; mTOR, mammalian target of rapamycin; OT, ovarian tissue; pAkt, phosphorylated Akt; PI3K/Akt, phosphatidylinositol 3-kinase/protein kinase B; UC, umbilical cord; VEGF, vascular endothelial growth factor.

In terms of follicle outcomes, co-transplantation with MSCs provided follicle pool protection, especially of primordial follicles (Xia *et al.*, 2015; Cacciottola *et al.*, 2020, 2021b; Cheng *et al.*, 2022), by both a decrease in follicle apoptosis (Xia *et al.*, 2015; Damous *et al.*, 2018; Buigues *et al.*, 2021b; Cacciottola *et al.*, 2021b; Cheng *et al.*, 2022) and PI3K/Akt pathway activation (Cacciottola *et al.*, 2021b). These findings may be explained by what is already known about the impact of MSCs on ovarian tissue revascularization. Indeed, earlier reoxygenation (Manavella *et al.*, 2018) and reperfusion (Xia *et al.*, 2015; Cacciottola *et al.*, 2021c) have been evidenced, along with greater revascularization of ovarian grafts (Xia *et al.*, 2015; Manavella *et al.*, 2018; Herraiz *et al.*, 2018a; Manavella *et al.*, 2019; Buigues *et al.*, 2021b; Cheng *et al.*, 2022). The main reason for this positive effect is the MSC secretome, which contains a number of proangiogenic factors like VEGF and bFGF (Manavella *et al.*, 2019; Buigues *et al.*, 2021a; Cacciottola *et al.*, 2021c).

Ovarian tissue transplantation outcomes are currently limited by massive follicle death occurring shortly after grafting because of both hypoxia-mediated apoptosis and non-physiological follicle activation (Dolmans *et al.*, 2021a). Transplantation approaches using MSCs to boost early graft revascularization may well address this issue in a clinical setting and enhance transplantation results. All patients would potentially benefit, but especially those with lower chances of fertility restoration using this technique. This includes subjects showing signs of an already depleted follicle reserve in their cryopreserved ovarian cortex, or with a damaged pelvis caused by repeated surgery or previous pelvic irradiation, making it unable to properly host ovarian grafts (Dolmans *et al.*, 2021b).

### Ongoing clinical applications

Use of MSCs to increase the likelihood of pregnancy in postmenopausal patients has already been recorded in a clinical context, with publication of case reports and small case series. The first live births were obtained by intraovarian BM-MSC injection by Edessy *et al.* (2016) in 1 out of 10 treated patients with POI (26–33 years), by Gabr *et al.* (2016) in 1 out of 30 treated patients with POI (18–40 years), and by Gupta *et al.* (2018) in one patient aged 45 years. Preliminary results on DOR subjects were published by Herraiz *et al.* (2018b) and the study is still ongoing. Seventeen patients with DOR (<39 years) undergoing intra-arterial catheterization for MSC mobilization using GCS-F were included. An improvement in ovarian function was observed in around 80% of patients within 4 weeks. There were increased numbers of antral follicles, particularly in the infused ovary, and also retrieved oocytes, with a significant drop in cancelation rates after controlled ovarian stimulation (Herraiz *et al.*, 2018b). Five pregnancies were obtained, three of which resulted in a healthy baby at the time of publication. The same group (Herraiz *et al.*) recruited 20 patients with POI (<39 years of age) for a new study investigating both intra-arterial catheterization and stem cell mobilization in peripheral blood using granulocyte-colony stimulating factor. The second technique relying on peripheral blood, which is much less invasive than the original approach, appears to have a systemic effect on both ovaries after stem cell injection, thanks to their homing ability to distant damaged sites, as previously suggested by several animal studies (Polonio *et al.*, 2020). Only preliminary data have been published so far, reporting menses recovery in around 40% of patients and one pregnancy to date (Polonio *et al.*, 2020).

Clinical application of sources other than BM-MSCs for fertility restoration in patients with POI have also been published recently. One study reported menses recovery in four out of nine patients (29–39 years) treated by intraovarian injection of AT-MSCs (Mashayekhi *et al.*, 2021). Another study reported menses recovery and increased antral follicle count in 4 out of 15 POI patients treated with autologous Men-MSCs (Zafardoust *et al.*, 2023). In terms of fertility restoration, four live births were obtained after treating 61 patients with POI (<35 years old) with UC-MSCs (Yan *et al.*, 2020).

All published studies have reported live birth rates of around 10% in patients with POI. These may be considered comparable to spontaneous pregnancy rates of around 5–10% in women with POI in the first few years after their diagnosis, owing to spontaneous but temporary ovarian reactivation (Fraison *et al.*, 2019). While these results are promising and corroborated by sound evidence from animal studies, ovarian rejuvenation with MSCs has not yet delivered the expected results. Future studies need to focus on ways of enhancing fertility in these patients (Rosario and Anderson, 2021). One way may be maximizing the effect of stem cell use by choosing the best conditions, in terms of cell type, concentration, and administration mode, in order to boost their biological impact on the dormant ovarian reserve. Another way may be advancing our understanding of the pathophysiology of POI in order to be able to select the subgroup of patients with the best chance of benefiting from this technique.

## Discussion/conclusions

The present article offers a comprehensive overview of possible future applications of MSCs in reproductive medicine. MSCs from different sources, including fetal and adult tissues, have been tested in different conditions, as have their derivatives (exosomes and the secretome contained in culture medium). The majority of studies consider follicle recovery after CHT exposure, with numerous *in vitro* studies on GCs and *in vivo* studies in murine models. All of them investigate the potentially beneficial impacts of MSCs on the follicle pool during or immediately after gonadotoxic damage, providing more in-depth knowledge of the ability of GCs to recover, as well as the dynamics governing follicle death and abnormal activation after injury. This model does not, however, represent the clinical situation of young women diagnosed with DOR or POI caused by gonadotoxic therapy years prior. This could be the reason for the discrepancy between outcomes of preclinical studies, which clearly show significant protection of the follicle pool mediated by MSCs, and results from the first clinical trials, in which the MSC effect appears to be much more modest. Further studies are needed to better understand the impact of MSCs on the follicle pool, particularly on oocyte quality, which may be the limiting factor responsible for the disappointing results.

Other promising clinical applications involving MSCs are emerging from the literature, in our quest to enhance fertility outcomes. Ovarian tissue co-culture with MSCs, used as feeder cells to improve follicle survival, growth, and oocyte competence, may serve to propel the ovarian tissue *in vitro* culture technique toward legitimate clinical application. Similarly, MSCs have also proved effective at boosting revascularization of the grafting site in the context of ovarian tissue transplantation. Indeed, data from preclinical studies using human ovarian tissue xenografting models appear robust and reproducible, suggesting a possible role for MSCs in counteracting large-scale ovarian follicle pool loss after grafting, which is still one of the main limiting factors of the technique.

To sum up, all gathered data on the one hand show that regenerative medicine techniques are quickly gaining ground among the innovative techniques being developed for future clinical application in the field of reproductive medicine. On the other hand, there is still a lot of work to do before MSCs can be safely and effectively used to improve follicle outcomes in different clinical applications. We are moving in the right direction but need to delve deeper to advance our fundamental understanding of these multipotent cells.

## Data Availability

The data underlying this article are available in the article text and in its tables.
